# The effect of erythropoietin to pulmonary injury and mast cells secondary to acute pancreatitis

**DOI:** 10.1186/1756-0500-7-267

**Published:** 2014-04-24

**Authors:** Tanzer Korkmaz, Nurettin Kahramansoy, Ali Kilicgun, Tulin Firat

**Affiliations:** 1 Department of Emergency, Medicine of Faculty, Abant İzzet Baysal University, Bolu, Golkoy, Turkey; 2 Department of General Surgery, Medicine of Faculty, Abant İzzet Baysal University, Bolu, Turkey; 3 Department of Thoracic Surgery, Medicine of Faculty, Abant İzzet Baysal University, Bolu, Turkey; 4 Department of Histology and Embryology, Medicine of Faculty, Abant İzzet Baysal University, Bolu, Turkey

**Keywords:** Erythropoietin, Acute lung injury, Mast cell, Acute pancreatitis

## Abstract

**Background:**

Acute pancreatitis is a life-threatening necroinflammatory disease that is characterized by systemic inflammatory response syndrome and acute lung injury even in its very first days. Erythropoietin (EPO) is a hormone considered as an antiapoptotic and cytoprotective with observed receptors of anti-inflammatory effect on organs apart from the liver and the kidneys. In this study, the effects of EPO on pulmonary mast cells and on secondary injury caused by acute pancreatitis are investigated.

**Methods:**

Twenty one Wistar Albino rats were divided into three groups—sham, control, and EPO groups—with 7 rats per group. Pancreatitis was induced by administering 4.5% sodium taurocholate into the pancreatic duct. A 1000 U/kg/day dosage (three times) of EPO was administered to the EPO group. Blood urea nitrogen (BUN), creatinine, amylase, and troponin I in the serum were studied; and lung, kidney, brain, and heart tissues were examined histopathologically.

**Results:**

There were no histopathological changes in the other organ tissues except for the lung tissue. Compared to the control group, the EPO group showed significantly reduced alveolar hemorrhage, septal neutrophil infiltration, lung wall thickness score, and mast cell count in the lung tissue.

**Conclusions:**

Administration of EPO reduces the mast cell count and lung wall thickness, and it reduces the alveolar hemorrhage and septal infiltration induced by acute pancreatitis.

## Background

Acute pancreatitis (AP) is a life-threatening necroinflammatory disease. Systemic inflammatory response syndrome (SIRS) that develops during the course of the disease and owing to multiple organ failure (MOF) is a major cause of morbidity and mortality. A large number of patients lose their lives due to acute lung injury (ALI) and acute respiratory distress syndrome (ARDS) [1]. The disease confines itself to a single organ 80% of the time; however, with the development of MOF, it is related to mortality by 40% [2,3]. Although supporting therapies have been developed for the treatment of pancreatitis, definite treatments capable of reducing the severeness of the inflammation do not yet exist [4].

The erythropoietin (EPO) gene is located on chromosome 7 and encodes a polypeptide chain containing 193 amino acids. The circulatory mature protein is a 30.4-kDa glycoprotein that has a span of 165 amino acids [5]. EPO is a hormone mainly produced in the kidneys that is effective in maintaining the erythrocyte mass in circulation. Furthermore, recent studies have shown that in addition to the liver and the kidneys, EPO and its receptors are also present in organs such as the heart and the brain [5]. By its receptors, EPO, as a cytokine has antiapoptotic and cytoprotective effects [5-7].

Although mast cells are present in all body tissues, they are mostly observed around the capillaries of the skin and the respiratory system and the vessels of the lymphatic system [8,9]. It has been reported that mast cells play a role in inflammatory and allergic diseases of the respiratory system [10-12]. In ischemic injuries, ALI fundamentally develops through the release of mast-cell-activated cytokines such as histamine and tryptase [12,13]. It is also reported that mast cells increase inflammation within seconds in lung tissue through these cytokines [9,13].

In this study, it is aimed to investigate histopathological effects of EPO administration on the tissue in distant organ damage induced by AP and variations in the mast cell count.

## Methods

This study was approved by the ethics committee of Abant İzzet Baysal University (No. 375/2012). Twenty-one healthy male Wistar Albino rats (weight: 150–200 g) were housed in a climate-controlled facility and given ad libitum access to food and water.

### Experimental groups

21 rats were randomly separated into three groups—sham, control, and EPO (with induced AP and application of EPO) groups—with 7 rats per group. After the inducement of pancreatitis, a dosage of 1000 U/kg/day (5 IU) of intraperitoneal EPO (Eprex, 2000 U/0.5 mL from Gürel İlaç Ticaret, İstanbul) was administered for three days to the EPO Group.

### Study Protocol

All interventions for rats were performed by the same team. In a temperature-controlled room, anesthesia was induced by intramuscular injection of a combination of xylazine hydrochloride (10 mg/kg, Rompun, Bayer, Toronto, Canada) and ketamine hydrochloride (50 mg/kg), Ketalar, Parke Davis-Eczacıbaşı, İstanbul, Turkey). The rats were fixed in a supine position and secured in the dissection tray. Their skin was shaved with povidone-iodine solution and prepared for aseptic surgery. The intestinal region was accessed by means of midline laparotomy, and the duodenum was pulled downward and laterally. The pancreatic duct was exposed and entered transduodenally using a 26G catheter. The sham group was administered 0.2 cc serum physiologic through the pancreatic duct, and the control as well as the EPO groups were administered a solution of 0.2 cc serum physiologic dissolved in 4.5% sodium taurocholate (NaTC) ((Sigma-Aldrich, St. Louis, MO, USA) through slow infusion into the pancreatic duct [14]. The transduodenal bile duct and anterior abdominal walls were closed with 6/0 prolen and 3/0 silk suture, respectively. After the procedure, the EPO group was administered 1000 U/kg EPO intraperitoneally at intervals of 1, 24, and 48 h, and the sham and control groups were administered the same volume of serum physiologic intraperitoneally. At the end of the third day, after sedation, all subjects were sacrificed by exsanguination, and the heart, kidney, brain, and pulmonary segments were removed. The blood, blood urea nitrogen (BUN), creatinine, amylase, and troponin I were examined.

### Biological examination

After the procedure, biochemical analyses were performed on blood samples taken from the abdominal aorta. BUN, creatinine, and amylase tests were performed using COBAS Integra 800 autoanalyzer (Roche Diagnostics GmbH, D-68298 Mannheim, Germany); and troponin I tests were performed using Mini Vidas analyzer (Biomerieux). Analyses for BUN were performed via the kinetic method using urease and glutamate dehydrogenase; those for amylase were performed via the calorimetric enzymatic method, and those for troponin I were conducted via the enzyme-linked fluorescent immunoassay method.

### Histopathological evaluation

Lung, brain, heart, and kidney tissues taken from the subjects were fixed in 10% neutral formalin. Sample tissues were submerged in paraffin after being put through degreed alcohol and xylene. 5-μm-thick sections were taken and dyed with hematoxylin eosin and toluidine blue for general examination and for obtaining the mast cell count, respectively; and they were assessed using a BH2 Olympus light microscope. Histopathological evaluations were performed on each subject’s lung, brain, heart, and kidney sample sections. Changes in the lung tissue were scored in terms of alveolar edema, hemorrhage, cell infiltration, and alveolar wall thickness. Scoring was assigned as normal structure (0), minor changes (1), moderate changes (2), and widespread changes (3) [15]. In the lung tissue of all subjects, the mast cell count was obtained at 40× scope magnification in 6 different areas. Histopathological scoring was performed by a researcher who was not informed about the subject groups.

### Statistical analysis

The SPSS software package (SPSS version 17 for Windows) was used for statistical analyses. The values were presented as mean ± standard deviation (SD), which is the normal distribution of the data, and as median ± standard error (SE), which is not the normal distribution of data. For comparison among the groups, Kruskal-Wallis variance analysis, and for binary analysis, Mann-Whitney test with Bonferroni correction, were used. These differences were considered significant when probability was less than 0.05.

## Results

All subjects survived during the study process. Biochemical analysis revealed higher values of amylase in the control (2747 ∓ 1288,8) than in the other groups (group S:2182 ∓ 0, group EPO:1931 ∓ 420,4). The troponin I value was found to be <0.001 ng/mL in all rats in the sham group, <0.001 mg/dL in four rats and 0.5–22.97 ng/dL in five rats in the Control group, <0.001 ng/dL in two rats and 0.2–0.9 ng/dL in all the remaining rats in the EPO group. The intergroup analyses showed no significant variations for amylase, BUN, and troponin I values but a statistically significant difference for the creatinine value (p = 0.001) was. Binary comparisons showed that the difference in creatinine value was due to the Control (Median ∓ SE: 0,3 ∓ 01) and the EPO groups (Median ∓ SE: 0,2 ∓ 0,005) (p = 0.001).

### Histopathological results

**Figure 1:**
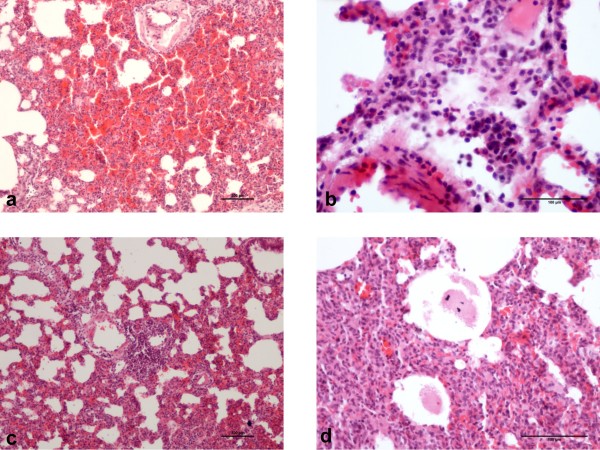
Histologic changes in the control group: (a) Extensive hemorrhage in lung parenchyma, (b) inflammatory cells neutrophil infiltration, (c) vascular proximity lymphocyte infiltration, (d) alveolar fluid (hematoxylin eosin-x40).

**Figure 2:**
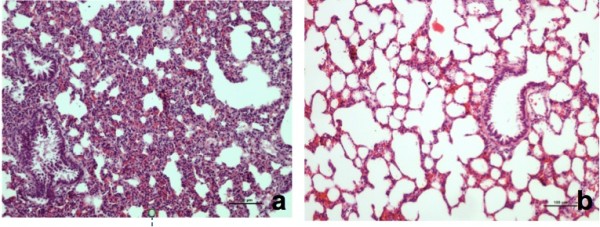
Histologic changes in the EPO and Sham groups: (a) reduced hemorrhage in the EPO group (hematoxylin eosin-x40), (b) histological appearance of the lung in the sham group(toluidine blue dye).

No structural changes were observed in histopathological examinations of tissue samples taken from the kidney, brain, and heart but the lung. In the lung, parenchyma of control group subjects, capillary saturation and hemorrhage, inflammatory cell infiltration with accumulation of neutrophils, lymphocyte cell concentration around vessels, thickening of interalveolar walls, and fluid within some alveoli were identified (Figure 1a,b,c,d). Histological changes in the control and sham groups were also shown in Figure 2.

**Figure 3:**
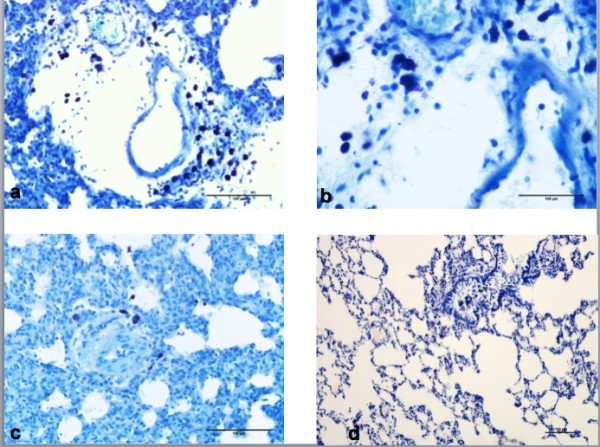
Distribution of mast cells in the groups: (a) Pronounced edema and a multitude of mast cells in vascular proximity of control group, (b) at larger magnification, the majority of mast cells are seen to be degranulated (c) decreased mast cell count and edema in EPO group (toluidine blue dye), (d) rare mast cells in the sham group (toluidine blue dye).

It was observed that in the subjects of the Control Group there were mast cells with spread granules in some areas; and in these areas edema was very significant (Figure 3a,b). In the EPO group, in addition to the mast cell count surrounding the vessels, hemorrhage and edema were observed to be reduced (Figure 3c). And the presence of scant amount of mast cells in sham group was shown in Figure 3d.

The scoring used in the histopathological assessment and the observed histopathological changes including alveolar congestion, hemorrhage, alveolar septal infiltration, and alveolar wall thickness are presented in Table 1 and Figures 1 and 3. There was no statistically significant difference in terms of alveolar congestion (p = 0.368) in between-groups comparison. When hemorrhage, alveolar septal infiltration, and alveolar wall thickness scores were evaluated, a meaningful intergroup difference was found. In further analysis, a meaningful difference was found between the sham and the control groups, the sham and the EPO groups, also the control and EPO groups (p = 0.001, 0.002, and 0.019, respectively) in terms of hemorrhage. A similarly significant difference was observed in terms of both the alveolar septal infiltration and the alveolar wall thickness between the sham and the control groups in addition to the sham and the EPO groups (p = 0.001 in both cases).

**Table 1:** Evaluation of the histopathologic changes in the lung

**Lung injury score**	**Groups [median (min-max)]**			**p**
	**Sham**	**Control**	EPO*	
Alveolar congestion	0	1 (0-1)	0	0,368
Hemorrhage	0	2 (1-2)	1 (0-1)	0,001
Alveolar septal infiltration	0	2 (1-2)	1 (1-1)	0,001
Alveolar wall thickness	0	2 (1-2)	1 (1-1)	0,001

Histopathological score: “0” normal, “1”mild, “2”moderate.

*Group EPO: Erythropoietin therapy group.

The median count of mast cells in lung tissue samples was determined as 8 ± 0.5 in the sham group (95% CI, 7.1–9.7), 12 ± 0.8 in the control group (95% CI, 10.5–14.8), and 9 ± 0.3 in the EPO group (95% CI, 8.2–9.7). In the control group, pronounced edema and a large number of mast cells with spread granules surrounding the vessels were observed (Figure 3a,b). On the other hand, in the EPO group, in addition to the mast cell count surrounding the vessels, hemorrhage and edema were reduced (Figure 3c). In terms of mast cell count, a significantly difference was found (p = 0.001) between the groups. Further analyses showed that this difference was due to the Control and the EPO groups (p = 0.002). The mast cell count was observed to be significantly lower in the EPO group compared to the control group.

## Discussion

Although AP is an organ disease, it can cause systemic diseases in other organs such as the lungs, heart, kidneys, and brain as well. In published case presentations, acute myocardial infarction has been identified in patients with AP. In these patients, despite the ST-T changes in ECG, enzyme variations, troponin I values, and post-angiography coronary arteries are found to be normal. It is attributed to the direct effect of proteolytic enzyme concentrations, vasoactive and toxic substance release, vasovagal reflex, hypovolemia, electrolytic changes, and fat emboli on heart muscles [16-18]. In this study, no meaningful difference was observed in terms of troponin I values and histopathological assessments among rat groups with induced AP.

Acute renal failure is generally observed as a lethal complication of AP [19-21]. In this study, only the creatinine value of the kidney in the EPO group was found to be significantly lower than that of the control group. No changes were identified in terms of histopathology.

Among new treatment strategies, EPO therapy to retard the course of neurodegenerative diseases and slow down their damaging effects is considered promising [6,22,23]. Studies have already focused on the use of low molecular weight heparin, dexamethasone, and octreotide for encephalopathy developed in AP-induced rats [24,25]. However, as studies have not yet focused on EPO use for brain damage caused by induced AP, it is currently not possible to reach a conclusion on this issue. In this study, no histopathological changes were seen in the brain tissues of rats with induced AP. However, further studies may produce more detailed data on the inhibitive effect of EPO and secondary cerebral damage caused by AP.

SIRS and MOF are the most frequent causes of mortality in AP. Organ failures are considerably affected by pulmonary complications. Three major pathological mechanisms have been identified in ALI induced by pancreatitis: (1) pulmonary endothelial dysfunction, (2) leucocyte migration in interstitial area, and (3) distortion of wall permeability and gas exchange due to morphological pulmonary damage [10,26].

Pancreatitis-related ALI (PALI) is seen as one of the causes of mortality in the initial days of the disease [10]. Renner et al., through a study of 405 case autopsy examinations, identified that 60% of patients had pulmonary complications in the first week [27]. Johnson et al. identified pulmonary complications in 38–39% of patients [28]. Tascilar et al., by EPO (1000 U/kg, single dosage) therapy, identified a meaningful reduction in alveolar edema and polymorphonuclear cells (PMNL) in ALI induced by pancreatitis after 72 h [29]. Li et al., in the treatment of AP, and Shang et al., in the treatment of lung injury caused by lipopolysaccharide, applied a single dose of 3000 U/kg EPO prior to the procedure. In both cases, positive histopathological results were obtained. Meaningful reduction in the lung tissue interstitial edema, hemorrhage, thickening of the alveolar wall, alveolar area, and inflammatory cell infiltration were identified in the EPO group [30,31]. This result was correlated with the anti-inflammatory effect of EPO. Although this study did not show a meaningful difference in terms of alveolar edema between the EPO group and the other groups, a meaningful difference was observed in neutrophils accumulated in alveolar septal cell infiltration in the EPO group compared to the control group. In contrast to our study, two cited studies have applied EPO therapy with a single 1000 U/kg/day dose. The EPO dosage is related to the cytoprotective inhibition effect. Dosages provide systemic cytoprotection at 100–1000 U/kg, neuroprotection at 350–5000 U/kg, and cardioprotection at 3000–5000 U/kg [14,32].

In this study, a dosage of 1000 U/kg/day (third dose) was used to reduce inflammatory cytokines and protect the cells from apoptosis. Alveolar edema developed secondary to pancreatitis is connected with the increase in microvascular permeability [27]. It may be considered that EPO protects endothelial cell integrity and reduces lung damage [29]. The data show that EPO administration can alleviate pulmonary injury parameters in pancreatitis.

Mast cells are important due to their location around blood capillaries and the lymphatic system and their ability upon activation to cause allergic and inflammatory diseases of the pulmonary system [10,12]. In ALI, the increase in pulmonary neutrophil infiltration may be related to the extreme activity of macrophages and resulting chemotactical neutrophil migration, which cause the release of various inflammatory mediators. In the ALI development process, pancreatitis activates mast cells and then leukocytes, which lead to endothelial wall dysfunction [10]. In a lung ischemia reperfusion experiment in which rats were administered mast cell stabilizer agent (cromolyn sodium) prior to the procedure, a reduction in inflammatory response was identified [10,33]. In the histopathological assessment of this study, the mast cell count of the EPO Group (9 ± 0.3) was observed to be significantly lower than that of the control group. The findings also support the therapeutic effect of EPO in ALI treatment, similar to available literature data.

## Conclusions

EPO is effective in limiting lung damage secondary to AP. In this regard, it may be considered that it has positive effects in the reduction of mast cell count in the lungs, alveolar hemorrhage, septal neutrophil infiltration, and wall thickness. However, further studies are required to investigate the effects of EPO on the mast cells during ALI.

## Competing interests

The authors declare that they have no competing interests.

## Authors’ contributions

All authors contributed equally to the writing and revision of the manuscript. All authors read and approved the final manuscript.
